# Development, implementation, and evaluation of a flagship simulation-based capstone course for graduating medical students in the Middle East

**DOI:** 10.3389/fmed.2025.1684952

**Published:** 2025-11-12

**Authors:** Zakia Dimassi, Mohammed Abuzitoon, Masood Ahmad, Dana Lutfi, Thripti Vijayakumar, Nora Kakati, David Murray, Salman Guraya

**Affiliations:** 1 Department of Medical Sciences, Khalifa University College of Medicine and Health Sciences, Abu Dhabi, United Arab Emirates; 2 Khalifa University College of Medicine and Health Sciences, Abu Dhabi, United Arab Emirates; 3 Sheikh Shakhbout Medical City, Abu Dhabi, United Arab Emirates; 4 Washington University, Saint Louis, MO, United States; 5 Department of Clinical Sciences, College of Medicine, University of Sharjah, Sharjah, United Arab Emirates

**Keywords:** transition to residency, capstone, international medical graduates, EPA, simulation-based education, e-learning

## Abstract

**Background:**

Transitioning from undergraduate to graduate medical education is characterized by challenges related to clinical competence, professional identity formation, and the adoption of system-based practice. This transition serves as an accountability measure for medical schools, particularly for international medical graduates (IMGs). Unfortunately, there remains a gap in medical education that may compromise the fitness to practice of graduating doctors. To address this gap, this work aimed to develop, implement, and evaluate a simulation-based Transition to Residency (TTR) capstone course within a Doctor of Medicine (MD) course that aligns with the Entrustable Professional Activities (EPAs) and specifically targets the educational shortcomings experienced by new medical graduates.

**Methods:**

Our course adopted the modified Kern 7-step approach and incorporated simulation-based, Kolb’s experiential learning, and e-learning constructs. The core themes included patient safety, basic and advanced procedural skills, “night on call,” acute case management training, and life support training. The main themes were mapped to high-yield competencies that corresponded with the core EPAs. A structured study plan, clear learning objectives, assessment tools, and full integration of educational and simulation technologies were developed. The pre- and post-data regarding students’ self-assessment of competence, their performance assessment (Kirkpatrick’s level 2), and their satisfaction with the learning outcomes (Kirkpatrick’s level 1) were analyzed.

**Results:**

The success of this course was evident in the high student satisfaction rates and the overall increase in their self-assessment of skill acquisition across domains in all 3 years, with persistently highest improvements in the psychomotor domain (*p* < 0.001 and Cohen’s d = 1.02, 2.29, and 1.44) compared to cognitive and communication domains. From a course organization standpoint, centralizing communication, appointing independent assessors, managing workload, and digitizing all procedures mitigated several challenges faced.

**Discussion:**

Our study highlights systematic implementation strategies, potential challenges, sustainability concerns, and future recommendations of a flagship capstone course, including the development of residency-specific training options. The high satisfaction rates and documented enhancement in all competency domains of the capstone course affirm its role in bridging gaps in medical education.

## 1 Introduction

The transition from undergraduate to graduate medical education (UME-GME) is a hallmark event where new graduates begin to provide unsupervised care and navigate the complex healthcare ecosystem, all while striving to maintain a healthy life-work balance and lifelong learning. Medical schools are committed to preparing their new medical graduates for clinical practice, thereby mitigating healthcare-related adverse outcomes for patients, as well as for physicians who have been facing alarmingly high rates of burnout (1–4). Readiness for the UME-GME transition is based on entrustment decisions and serves as a proxy for the degree of consistency and robustness of the clinical learning experiences. Entrustment, as defined by the Association of American Medical Colleges (AAMC) core Entrustable Professional Activities (EPAs) (5), demands more curated training and rigorous assessment data. Notwithstanding, the unpredictability of the clinical workplace, coupled with the nature of workplace-based education where multiple-level learners are engaged simultaneously, has always posed a challenge to the educational process. On the one hand, learners must accumulate the necessary clinical exposure and experience in their competency domains. On the other hand, clinical educators struggle to deliver congruent developmental levels of teaching and assessment, ensuring equitable learner participation in clinical workplace activities (6, 7). Moreover, reports from medical graduates suggest limited and lower-quality clinical experiences, supervision, and feedback (8), as well as substantial variations between clinical clerkships and individual students (9).

Emerging evidence of gaps in the competence of new medical graduates (10) and associated patient safety concerns (11–15) prompted a reevaluation of training approaches and assessment methods. The EPAs, first introduced in 2005 (16), shifted the focus of competency-based medical education to measuring units of real-world observable clinical activities. In parallel, preparatory UME-GME condensed courses were introduced into medical curricula, addressing the practical aspects of patient care, professionalism, patient safety, and deliberate practice of basic and advanced procedural skills. Dubbed as Transition to Residency (TTR), these courses have a proven track record of effectiveness in improving students’ confidence level in various skills (17), specifically when incorporating experiences such as reflections on successes and challenges faced and strategies for problem-solving. Primarily simulation-based, TTR experiences offer students opportunities to reinforce their knowledge, skills, and attitudes under the direct supervision of experienced professionals in a safe and controlled experiential learning environment. The resulting rigorous assessment data provide reliable information about the students’ actual clinical abilities (18). Despite their importance, few scholarly publications exist on the rationale, instructional design, simulation modalities, and implementation of these transition courses (4, 19–22). The available publications also show a lack of standardization in the timing, duration, specificity, and assessment methods (23, 24).

The recent developments in the United States Medical Licensure Exams (USMLEs), starting with setting the USMLE Step 1 exam as pass/fail, followed by the abrupt cancellation of the Step 2 Clinical Skills (Step 2 CS) exam during the COVID-19 pandemic, compounded the problem of inadequate assessment data. International medical graduates (IMGs), physicians practicing medicine in a country different from their country of primary medical qualification (25), were particularly impacted by these changes, as they were denied the competitive edge of high Step 1 scores (26) and the opportunity to demonstrate their cognitive, communication, and psychomotor skills in Step 2 CS (27, 28). Concomitantly, major concerns arose about graduating “substandard” physicians, stressing the need for establishing “valid, reliable, fair, feasible, verifiable, appropriately delivered, and managed competency-based assessment” (29) to replace Step 2 CS. Expert recommendations, therefore, prompted the enabling of rigorous local clinical assessments to support all clinical skills EPAs, as well as learning activities encompassing different encounter formats and skill domains (18). More publications followed, suggesting UME-GME capstone course topics (30, 31).

As the first of its kind in the Middle East and North Africa (MENA) region, the capstone course at the Khalifa University College of Medicine and Health Sciences (KUCMHS) Doctor of Medicine (MD) program represents a flagship EPA-aligned and TTR curriculum-based course developed to support final-year medical students through a structured, simulation-integrated training. Its design aimed to fill the critical gap in regional medical education, particularly for IMGs, by offering a replicable and adaptable model for other high-performing academic institutions. By incorporating simulation-based education and leveraging a digital learning management system, the course was planned to foster clinical readiness and professional identity formation during the transition to postgraduate training (32). Given the novelty and regional significance of this course, this study seeks to explore how participation in a simulation-enhanced capstone course, aligned with EPAs, influenced final-year medical students’ self-confidence and overall readiness for independent clinical practice.

## 2 Pedagogical framework

We developed and implemented an intensive capstone course during the last month of the 4th year of the MD program at KUCMHS between 2023 and 2025. The primary aim of the course was to ensure integration of high-yield skills to prepare the medical graduates for transition to residency. Since its launch, the course has undergone enhancements based on students’ needs assessments and feedback, as well as observations by the organizing team. We present a detailed roadmap outlining the steps involved in course development, implementation, and improvement. This includes the course blueprint, organization, logistical and human resources management, content digitization using learning management systems, and incorporation of assessment tools for direct observation of performance. To evaluate the course effectiveness, we analyzed the students’ satisfaction, their performance metrics, and pre- and post-course self-confidence assessment. These detailed descriptions offer a comprehensive roadmap to medical educators involved in UME-GME transitions, particularly in international MD programs, to effectively implement a similar course in their curricula.

### 2.1 Settings and sample

This is a longitudinal descriptive study of a required capstone course, delivered over three academic years from 2023 to 2025, at the Center for Experiential Learning and Clinical Simulation (CELS) at KUCMHS, Abu Dhabi, United Arab Emirates. The course was scheduled for March and April and was positioned as the final course in the medical curriculum. Preparations for the course, including scheduling, identifying and sourcing external facilitators, and logistical planning, would begin 3 months before the course delivery. Participants included all 4th-year MD students enrolled in the KUCMHS MD program.

### 2.2 Ethical approval

The study was reviewed and approved by the Khalifa University Office of Research Services Compliance (#H23-043) as shown in Appendix 1. All learners signed the informed consent form and gave written permission for audiovisual recording for the purposes of simulation-based educational activities.

### 2.3 Pedagogical framework and course design

The capstone course was designed following a model that integrated best practices for effective simulation-based training and a modification of Kern et al.’s 6-step approach for curriculum development (33) (Supplementary Table S1). We aligned the simulation-based sessions with Kolb’s experiential learning cycle (34). Given that our learners were final-year medical students with prior exposure to a broad set of clinical skills, they entered Kolb’s cycle at the stage of active experimentation rather than starting with concrete experience. The sessions’ learning objectives, contents, and assessment tools were aligned with the AAMC EPA framework (5).

### 2.4 Problem identification and general needs assessment

Setting USMLE Step 1 as pass/fail and canceling Step 2 CS during the COVID-19 pandemic altered the UME landscape. It compounded the pre-existing challenges of providing adequate, standardized, and structured clinical education amid the busy and often chaotic clinical workplace. Anticipating the impact of these interventions on our graduates’ educational experiences and their chances of matching into residency courses locally and in the US, a robust training course was deemed necessary.

### 2.5 Targeted needs assessment

Based on the identified gaps in the clinical training at the KUCMHS, the results of the workplace-based assessment of the students, and Bandura’s social cognitive theory (35) of self-efficacy, we devised a generic survey in 2023, followed by a more in-depth needs assessment survey. The questions were formulated to reflect the students’ perceived level of general self-entrustment and self-assessment of competence on high-yield skills necessary for independent, safe healthcare delivery. The results helped us refine the learning objectives and schedule the sessions.

### 2.6 Goals and objectives

**Figure 1:**
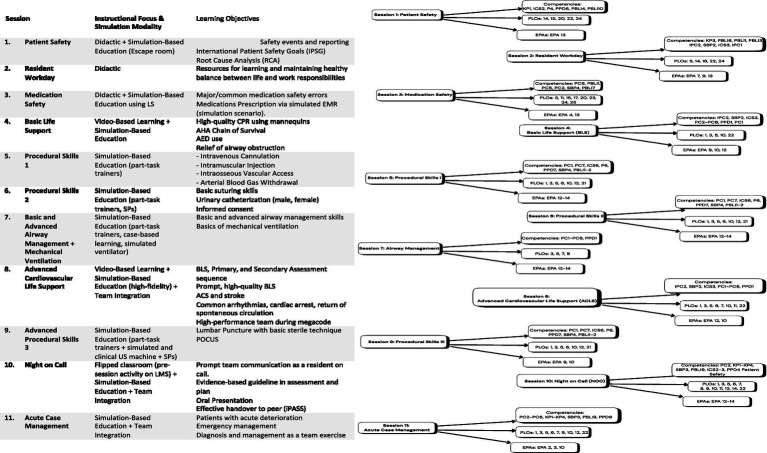
Capstone complete mapping of EPAs, competency domains, and CLOs (right); capstone sessions, learning objectives, instructional focus, and simulation modality (left).

The primary goals of the capstone course were to provide structured high-yield training experiences that would potentially improve the readiness of the KUCMHS graduating MDs for independent clinical practice. The course was conducted in a safe simulation-based learning environment that fosters direct supervision and real-time feedback on performance from subject matter experts. To ensure extensive alignment across the curriculum, a blueprint of the core EPAs, competency domains, learning objectives (LOs), and assessment tools was designed (Figure 1; Supplementary Table S2), with an emphasis on psychomotor, cognitive, and communication competencies.

## 3 Learning environment

### 3.1 Educational strategies

**Figure 2:**
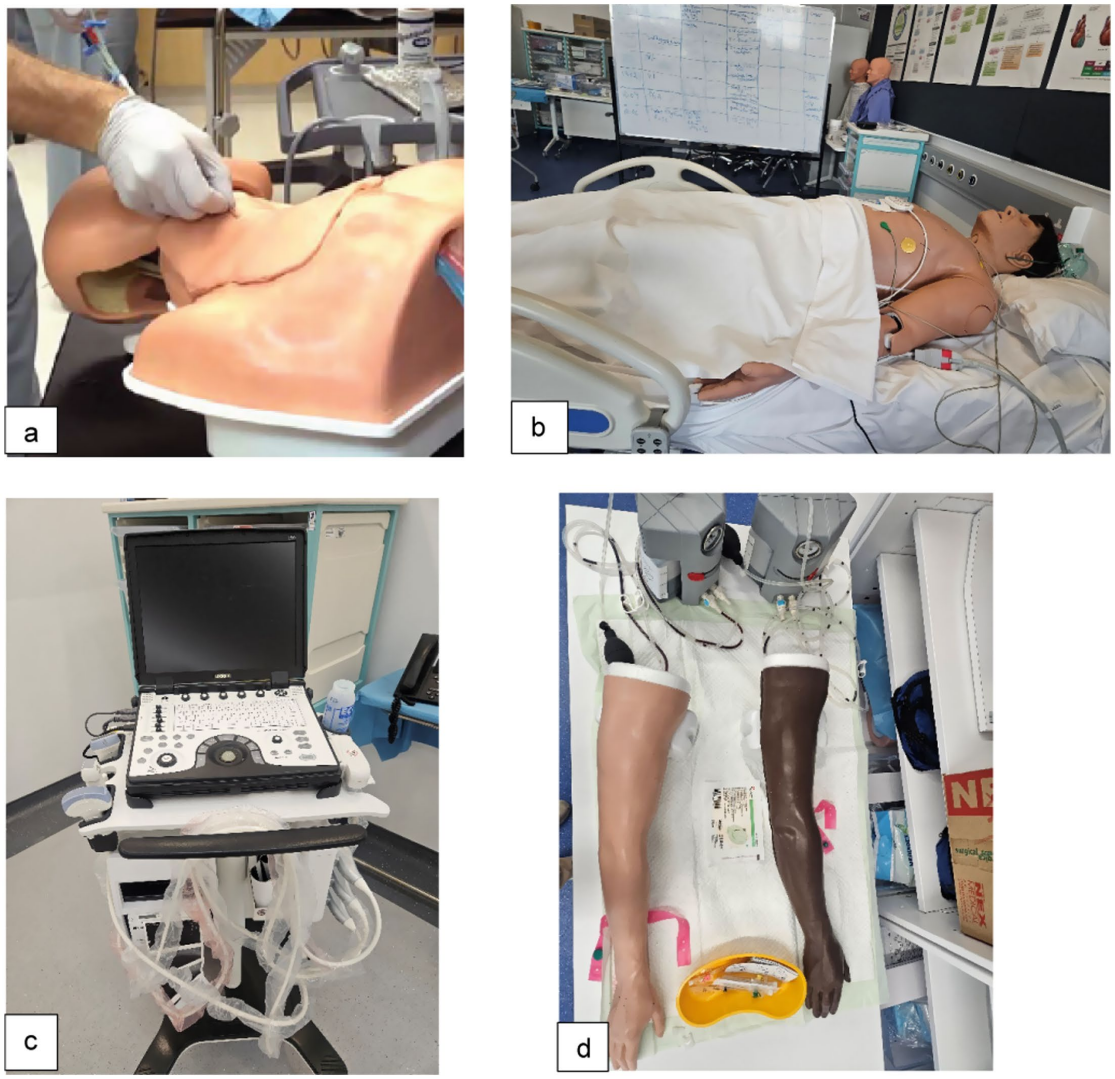
Various simulation modalities aligned with the sessions’ learning objectives. **(a)** Ultrasound (US)-guided central line insertion: part-task trainer with simulated US machine; **(b)** Advanced cardiac life support (ACLS): high-fidelity manikin. **(c)** Point-of-care ultrasound (POCUS): clinical US machine; **(d)** Intravenous (IV) line insertion: part-task trainer “IV arm.”

The organizing team of simulation experts collaborated with clinical subject matter experts (SME) to develop the sessions’ contents. Following a flipped classroom design, the students were assigned pre-session learning and assessment materials posted on the university’s learning management system (LMS), including Blackboard™ (Bb) and LearningSpace™. The pedagogical principles followed were learner-centeredness, simulation-based education, and deliberate practice (36–38) using various simulation modalities such as simulated patients (SPs), hybrid simulations, part-task trainers, and low- to high-fidelity manikins, aligning with the respective session learning objectives (4) (Figure 2). An adapted version of Night on Call (NoC) (39), a simulated learning experience that assesses near-medical graduates’ readiness for internship and entrustment judgments, was introduced as of Capstone 2024.

#### 3.1.1 Faculty, staff, and medical student preparation and pre-briefing

The primary capstone organizers (DL, DM, NK, and ZD) met weekly to discuss the planning of the sessions, and the capstone course was placed as a recurring agenda item within the weekly meetings. This practice facilitated the sharing of updates and enabled the tracking of assigned tasks and deliverables, which were critical to the course’s success. All external facilitators were provided with comprehensive briefings, initially conducted verbally over the phone, and subsequently formalized as a part of their formal invitation to participate in the course. This approach was aligned with the Society of Simulation in Healthcare (SSiH) accreditation standards, specifically, the Teaching/Education Accreditation Standards (40). Facilitators received calendar invitations that included important logistical information, such as a map of the KUCMHS main campus and the requisite dress code. Each session included an iteration of detailed instructions on Bb to orient the medical students. This encompassed a scheduling roster, which was later projected on a large TV screen in the simulation classrooms on the day (Supplementary Table S4). An in-person pre-briefing was conducted during the initial 10 min to clarify expectations, session objectives, the learning environment, and simulation modalities. Afterward, the students were divided into smaller groups for activities.

### 3.2 Individual assessment and feedback (Kirkpatrick level 2)

The degree of the students’ skill acquisition was measured as the change between the pre- and post-self-assessment of competence in the various skills, in addition to direct assessment of performance during each session.

At the outset and conclusion of the course, students’ self-assessments of their overall readiness for unsupervised practice and clinical competence in various high-yield skills were collected. The data from pre- and post-course self-assessments of competence were compared for evidence of improvement. A questionnaire for data collection was developed around the concept of self-assessment of entrustment and of skills competence necessary for postgraduate year 1 (PGY-1) residents. This step also served as a targeted needs assessment. The content validity was established by piloting the questionnaire amongst the students, which was then modified based on their feedback.

**Figure 3:**
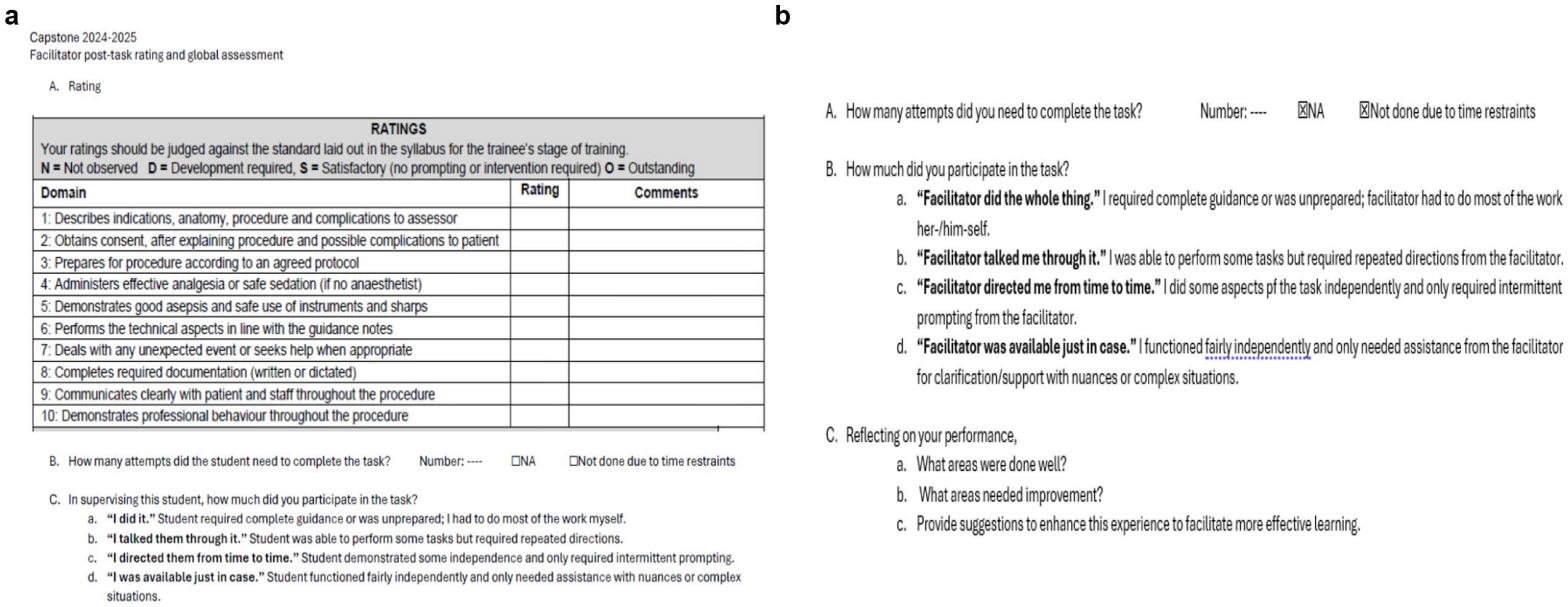
Direct Observation of Procedural Skills (DOPS) and Modified Ottawa Co-Activity Scale (MOCAS) assessment tools. **(a)** DOPS + MOCAS facilitator version is a hybrid assessment tool where facilitators scored student performance using the DOPS rating checklist and determined their entrustability based on the degree of needed supervision on the MOCAS. **(b)** The MOCAS student version is a self-assessment of entrustability where students rated the degree of supervision they needed to complete psychomotor skills.

The criteria for competence in procedural skills were determined through discussions among the organizing team and SMEs, considering the EPA framework. In 2025, a hybrid tool was introduced that was based on two validated and widely used assessment tools: the Direct Observation of Procedural Skills (DOPS) (41–43) to assess competence and the Modified Ottawa Co-Activity Scale (MOCAS) (44–46) tool to evaluate overall entrustment per skill. The assessment of students’ performance was conducted by eight assessors who had been trained in the use of the assessment tools. Inter-rater reliability was established through a pre-assessment calibration of the tools and shared mental model consensus, where the assessors met and discussed observable performance expectations (Figure 3a). To encourage the students to reflect on their performance (reflection-on-action), a separate tool targeted the learner’s self-assessment of entrustment (MOCAS – student version) (47) (Figure 3b). While there is overwhelming evidence showing that medical students tend to inflate their performance on clinical encounters and communication skills versus objective knowledge-based exams (48, 49), recent findings suggest consistency between student and faculty assessment results (50), particularly as students develop a sense of belonging and become more focused on learning in the context of formative assessment (51, 52). Based on the MOCAS, criteria for passing a given procedural skills station were set at *students functioning fairly independently with minimal intervention by the experts.* Observing the principles of assessment in competency-based medical education and assessment for learning (53), we set the criteria at a non-compensatory pass/fail while allocating time for deliberate practice to attain an acceptable level of performance.

### 3.3 Course evaluation (Kirkpatrick level 1)

In the first two iterations of the course, we collected students’ evaluations of each session individually. In 2025, we collected the evaluations weekly; they included questions that reflected the LOs of each session using a 5-point Likert scale (Supplementary Table S5).

### 3.4 Implementation

The capstone course was piloted in 2023 and continued during the academic years of 2024 and 2025. The course spanned three and a half weeks and comprised 11 three-hour interactive sessions with significant individualized attention and engagement. The planning and implementation of the course followed the Healthcare Simulation Standards of Best Practice™ Simulation Design (54). The KUCMHS simulation team led the coordination and execution of the space, logistics, communication with the external facilitators, set up and arrangement of the simulation space and modalities, assigning and training of the standardized patients (SPs), student attendance record keeping, tracking students’ engagement with the pre-session materials on the LMS, and collection of all assessment and evaluation data. Each year, a dedicated “capstone launch meeting” was held to discuss the schedule and communication plan, role assignments, potential hurdles, and contingency plans (Supplementary Table S6).

The course sessions were facilitated by a multidisciplinary team of simulation and SMEs from KUCMHS and selected clinical experts from different affiliated academic medical centers in Abu Dhabi, UAE (Supplementary Table S6). A remediation week was scheduled for students who were absent from one or more sessions.

As the class size increased by ~ 1.5 times (class of 2023: 23 students; class of 2024: 21 students; class of 2025: 31 students), the operational aspect of the course grew more demanding as the team strived to maintain the same educational quality, modalities (small group, hands-on, simulation-based) and assessment rigor according to the EPAs framework. The associated workload distribution proved challenging to estimate and had to be revisited based on staff input.

As a part of the continuous quality improvement process, a pre-course needs assessment was conducted. Based on the increasing number of students during the last 2 years and to address students’ needs, new sessions were added that covered EPAs 1, 4–6, and 9 (5), Basic Life Support (BLS) certification, and Advanced Cardiac Life Support (ACLS) certification. The direct observation assessment tools were refined to better align with the EPA framework. Similarly, during the academic year 2024, a reflective practice session was conducted amongst the organizing team to identify key areas for improvement of the course design, organizational mechanisms, and team dynamics. These reflections, along with the students’ course evaluations, prompted some modifications in 2025. First, the staff workload was redistributed based on a more precise estimate of time on task. Second, the focus of the medication safety session was changed to target medication prescription skills and entering medication orders. Third, a student self-assessment of the entrustability scale was added, which aligns with the recently introduced EPA 14, “Recognizes that assessment of performance leads to growth” (5, 55), as well as with the KUCMHS MD program’s learning objectives (PLOs) #18 and #24 (Appendix 1). Fourth, individual students’ assessments were assigned to trained assessors from the simulation team, allowing the facilitators to dedicate their time fully to demonstration and real-time feedback. Fifth, all course content was uploaded to Teams to centralize communication. Finally, all course assessments were digitized by integrating LearningSpace™ as a simulation platform into self-, peer-, and assessor evaluation of procedural skills and case-based learning activities (Appendix 3).

Following the Healthcare Simulation Standards of Best Practice, educational and simulation technologies were embedded throughout the capstone course instructional design, coordination, delivery, pedagogy, assessment, and evaluation. This provided an immersive, technology-enhanced environment for skill acquisition, clinical reasoning, and teamwork. Educational technology included Microsoft Teams™ as the central communication and collaboration platform for the course organizers, ensuring the timely sharing of course files and task coordination among team members. Blackboard™ was used as the primary LMS for posting course materials, announcements, and online interactive sessions (peer assessment of medication order entry in session 3). LearningSpace™, a cloud-based audiovisual (AV) recording system specifically designed for simulation-based education, facilitated scheduling, structured debriefing strategies (NoC), and digitizing skills assessments (medical error reporting in session 1; all procedural skills assessment checklists DOPS and MOCAS). Microsoft Forms™ was employed to collect pre-course self-assessments, post-course evaluations, and learner feedback, facilitating rapid data collection and analysis. Simulation modalities were integrated to align with the sessions’ LOs (Supplementary Table S1; Figure 2). These included simulated patients (SPs) for informed consent, hybrid simulations combining SPs with part-task trainers and/or clinical equipment for POCUS, and part-task trainers for procedural skills stations such as IV line insertion and LP. Low- to high-fidelity manikins were used in ACLS, acute care interventions, and interprofessional team responses as part of NoC.

Data analysis was performed using Jamovi (Version 2.6; The jamovi project, 2025). To evaluate the effectiveness of the intervention, the mean scores for each of the three domains were computed for pre- and post-intervention. A paired *t*-test was calculated to assess the significance of score change per domain. To quantify the magnitude of the observed effect for each comparison, Cohen’s d was computed with values interpreted as follows: small (0.2), medium (0.5), and large (0.8) (Cohen, 1988). A two-tailed alpha level of 0.05 was used as the threshold for statistical significance for all tests.

## 4 Results

Over the 3 years of the course implementation, most students reported satisfaction with the sessions’ learning outcomes (Supplementary Table S7), a proxy to the course’s value, relevance, quality of content, and performance of facilitators (Kirkpatrick Level 1) (56). The pre- and post-intervention analysis of the students’ confidence levels across various stations (Kirkpatrick Level 2) (56) affirmed a general trend of statistically significant improvements across nearly all domains and years, with effect sizes generally ranging from medium to very large. In 2023, significant improvements were observed in psychomotor (*p* < 0.001, d = 1.02) and cognitive (*p* = 0.031, d = 0.48) skills, but not in communication (*p* = 0.124), as this was not emphasized as a stand-alone domain. The 2024 cohort showed significant improvement with very large effect sizes in all three domains: psychomotor (*p* < 0.001, d = 2.29), cognitive (*p* < 0.001, d = 1.91), and communication (*p* < 0.001, d = 1.33). Similarly, in 2025, all domains demonstrated significant improvement: psychomotor (*p* < 0.001, d = 1.44), cognitive (*p* < 0.001, d = 0.76), and communication (*p* = 0.015, d = 0.46).

The analysis of the students’ assessment data of entrustability on procedural skills (IV, IM, IO, ABG, suturing, Foley catheter insertion, lumbar puncture) using the data from the capstone 2025 MOCAS revealed a non-statistically significant (*p* = 0.3372) discrepancy between the students’ self-assessment and the assessors’ assessment of entrustability. In their entrustment self‑assessments, some students acknowledged a need for direct supervision to execute particular skills. These results can inform targeted learning goals and the selection of suitable development activities.

## 5 Discussion

### 5.1 Objectives

The capstone course at KUCMHS is, to our knowledge, the first American-model TTR training activity implemented around the EPAs framework in the Middle East. We provided granular descriptions of the course design and continuous quality improvement processes while highlighting successes and challenges encountered, outlining a blueprint for replication in similar educational settings. The course was conceptualized to meet the educational and training needs of the first cohort of the KUCMHS MD graduates. Its primary intended outcomes included familiarizing medical students with the expectations of resident trainees, focusing on essential communication, cognitive and psychomotor skills, patient safety, and the balance between personal and professional obligations (3). Scheduled during the final weeks before graduation, the course was carefully designed to maximize relevance and benefits for students transitioning to graduate medical education. The curriculum was constructed around core EPAs (5) and principles of SBE, focusing on critical clinical skills for safe unsupervised healthcare provision. Ensuring the readiness of medical graduates in these competency domains is touted to reduce the levels of burnout, stress, and depression, as well as minimize the heterogeneity in new interns’ competencies at the onset of the PGY-1 year (57). The capstone course at KUCMHS proved to be a successful experience, as is evident in the statistically significant improvement in the students’ skills, with the improvement in psychomotor skills being the most prominent. Additionally, the sustained engagement of a diverse group of clinical experts facilitated effective course delivery.

As with any SBE activity, the resource-intensity of setting up a condensed course conducted in small groups and in a flipped classroom format was a major challenge. Estimating time demands on each team member proved difficult, which sometimes led to uneven workload distribution (Supplementary Table S4). To address this, a detailed blueprint was developed to streamline session requirements, including roles, logistics, and simulation modalities (Supplementary Table S3). Effective alignment of assessment with learning objectives was practically unfeasible due to facilitators’ inability to divide the contact time between demonstration and feedback, and filling in the assessment forms. Designating dedicated assessors from the simulation team was a proper solution to yield reliable data. However, it did not overcome the issue of resource intensity, limiting scalability of the course with larger student populations and/or less experienced staff. Transitioning to a paperless system streamlined communication, minimized redundancy, and simplified document management through custom pages created on LearningSpace™.

**Figure 4:**
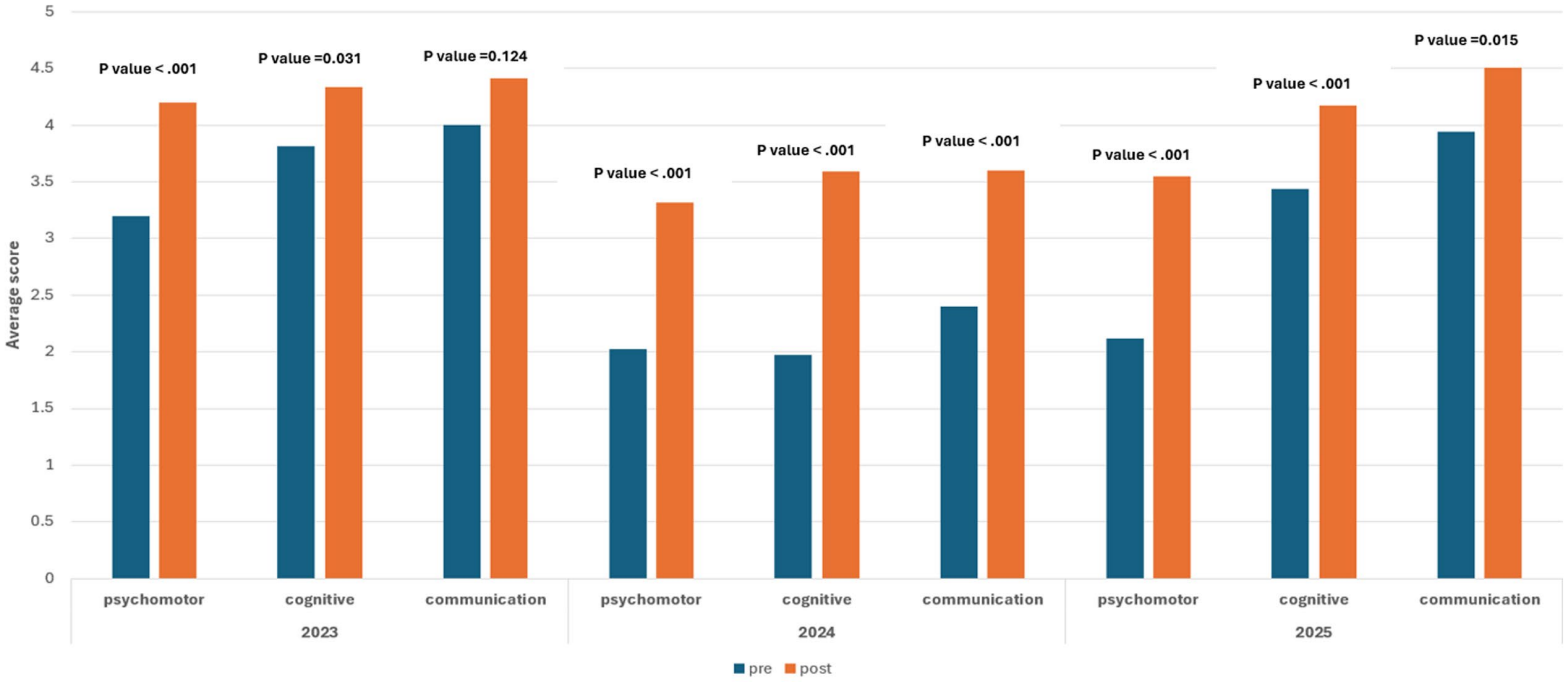
Graphic representation of significance in improvement and effect size across the three skill domains. Overall, significant improvement is observed across all domains over the 3 years, except for communication skills in 2023, as this domain was not given distinct emphasis.

The results of the students’ self-assessment of competence showed a clear distinction when comparing the pre- and post-course values in the psychomotor, cognitive, and communications domains. Overall, psychomotor skills exhibited the highest increase when compared to cognitive and communication skills (Figure 4; Table 1). This observation was expected, as the KUCMHS MD course offers a longitudinal and structured SBE that reinforces cognitive and communication skills throughout the 4 years of the program, starting with the Practice of Medicine course in Years 1 and 2. It also supports the notion that the acquisition of psychomotor skills benefits greatly from clinical simulation training. This modality has been shown to offer a more concentrated learning environment than traditional supervised clinical experience.

**Table 1:** Improvement trend in cognitive, communication, and psychomotor skills. Statistical calculations of significance of improvement and effect size across the three skills domains are presented and show significant improvements in all the years.

Year	Competency domains	Pre		Post		*p*-value	Effect size		
		Mean	SD	Mean	SD		Cohen’s d	95% CI of Cohen’s d	
2023	Psychomotor	3.2	0.55	4.2	0.91	<0.001	1.02	0.51	1.52
	Cognitive	3.81	0.57	4.34	1.04	0.031	0.48	0.04	0.91
	Communication	4	0.66	4.41	1.04	0.124	0.33	−0.09	0.75
2024	Psychomotor	2.02	0.47	3.32	0.44	<0.001	2.29	1.33	3.23
	Cognitive	1.97	0.39	3.59	0.66	<0.001	1.91	1.06	2.74
	Communication	2.4	0.62	3.6	0.78	<0.001	1.33	0.64	2
2025	Psychomotor	2.12	0.9	3.55	0.94	<0.001	1.44	0.92	1.95
	Cognitive	3.44	0.91	4.17	0.62	<0.001	0.76	0.36	1.16
	Communication	3.94	0.99	4.51	0.63	0.015	0.46	0.09	0.83

While the implementation of such a condensed high-impact course using SBE, small group learning, and systematic assessment based on direct observation of competence, is resource-exhaustive, it lays out an evidence trail of the well-thought-out and executed TTR preparation for our students. Together, such SBE-driven curricular interventions increase the students’ competitive edge and strengthen their residency applications, thus enhancing their employability in both national and international residency exams (58, 59).

### 5.2 Limitations

Although our capstone course at KUCMHS has demonstrated notable success, several considerations must be addressed. Firstly, the relatively small number of students may hinder the successful replication of this course in larger educational settings. Secondly, the resource-intensive nature of implementing the course in its current format poses challenges to its feasibility. Thirdly, the quality assurance process for assessment is particularly demanding, necessitating extensive training for assessors and the involvement of multiple dedicated evaluators in each session. Finally, the course evaluations aligned with Kirkpatrick’s levels 1 and 2 do not sufficiently provide evidence regarding the long-term benefits and the transfer of acquired knowledge to residency training.

### 5.3 Lessons learned

The capstone course at KUCMHS proved to be a successful experience, as is evident through the tangible results manifested in the statistically significant improvement in the students’ skills, with the improvement in psychomotor skills being the most prominent. The course articulates well with the UAE’s EmiratesMEDs (60), the country’s competency-based medical education (CBME) framework that is aimed at developing nationally approved standards for TTR courses. These courses should be informed by local residency programs’ needs assessment data to design residency-specific training offerings, with a focus on the psychomotor skills domain. The integration of structured TTR courses would also generate additional assessment data that feed into the programmatic assessment and students’ portfolios, further enriching input on the students’ performance relative to the target level of competence. The constraints related to the resource-intensive nature of course preparation, including feasibility, sustainability, and scalability, can be addressed through various measures that are applicable in any context with established CBME frameworks. TTR courses can be spread longitudinally across the clinical years, utilizing modest equipment. The tools would include high-fidelity mannequins obtained from local medical surplus stores that can be borrowed/rented, as well as a smartphone for recording. For sustainability and feasibility, pooling resources and the exchange of expertise across medical schools and simulation centers are viable options.

From a development and implementation perspective, educators are cautioned to thoroughly consider the feasibility of designing, organizing, and implementing such a resource-intensive course. While the outcomes showed improvement in students’ confidence levels, we suggest that a curated, specialty-specific training course would be a more attractive alternative that meets the variable learning needs of students, consolidating their level of performance in competencies necessary for the residency of their choice. Potential avenues to support the design and execution of TTR courses while optimizing cost and benefiting from emerging technologies include virtual simulation and automation of assessment using artificial intelligence specifically created for simulation LMS.

## 6 Acknowledgments of constraints

### 6.1 Conceptual constraints

While CBME and EPAs are well-established and validated frameworks in health professions education, a universal, shared mental model of the observable behavior that leads to a judgment of entrustability has yet to be attained. It is therefore challenging to confirm with certainty the reliability of entrustment decisions made by experts with diverse training backgrounds, who undoubtedly have differing understandings of the concept.

### 6.2 Methodological constraints

The research design and specific methods used to collect and analyze data were limited by the relatively small sample size and the relatively small volume of existing scholarly publications on the subject. The logistical constraints hindered hindered conducting long-term follow-up on the MD graduates’ transfer of skills into their residency training and future independent practice (Kirkpatrick’s levels 3 and 4). These can be addressed by assessing the transfer of learning of our MD graduates through personal interviews coordinated by the university’s alumni office, soliciting feedback on our graduates’ performance from the residency program directors at the institutions where our graduates match. As surrogate measures, we can examine our graduates’ scores on the National Institute of Health Specialties (NIHS) Emirates Medical Residents Entrance Examination (EMREE) and the USMLE Step 2 Clinical Knowledge (USMLE Step 2 CK), as well as their matching into US residency programs.

## Data Availability

The original contributions presented in the study are included in the article/Supplementary material, further inquiries can be directed to the corresponding author.
