# Correction: Development, implementation, and evaluation of a flagship simulation-based capstone course for graduating medical students in the Middle East

**DOI:** 10.3389/fmed.2025.1750655

**Published:** 2025-12-09

**Authors:** Zakia Dimassi, Mohammed Abuzitoon, Masood Ahmad, Dana Lutfi, Thripti Vijayakumar, Nora Kakati, David Murray, Salman Guraya

**Affiliations:** 1 Department of Medical Sciences, Khalifa University College of Medicine and Health Sciences, Abu Dhabi, United Arab Emirates; 2 Khalifa University College of Medicine and Health Sciences, Abu Dhabi, United Arab Emirates; 3 Sheikh Shakhbout Medical City, Abu Dhabi, United Arab Emirates; 4 Washington University, Saint Louis, MO, United States; 5 Department of Clinical Sciences, College of Medicine, University of Sharjah, Sharjah, United Arab Emirates

**Keywords:** transition to residency, capstone, international medical graduates, EPA, simulation-based education, e-learning

“Author Mohammed Abuzitoon was erroneously omitted as equal contributing author. The statement ‘These authors have contributed equally to this work' has been added to authors Zakia Dimassi and Mohammed Abuzitoon.”

There was a mistake in the supplementary files as published. The published content still had track changes and was not consolidated. Supplementary files Tables 1 and 2 have been replaced with supplementary file Table 1 which has all the supplementary material content. The supplementary file has been updated in the original article.

The original version of this article has been updated.

