# Supplementary material for: Development, implementation, and evaluation of a flagship simulation-based capstone course for graduating medical students in the Middle East
Source: Front Med (Lausanne). 2025 Nov 12;12:1684952. doi: 10.3389/fmed.2025.1684952 (PMC12647008; doi:10.3389/fmed.2025.1684952)
Supplement: Supplementary file 1 [file Table_1.DOCX]

| Table 1. Capstone course design following the modified Kern’s 6 steps | |
| --- | --- |
| Curriculum Development Steps | Capstone |
| 1. Problem identification and general needs assessment  a. Problem characterization  b. Current approach  c. Ideal approach  d. Gap analysis | - Changes in USMLE: Step 1 set as P/F, Step 2 CS canceled, IMGs most negatively impacted à medical programs graduating IMGs needed a replacement as evidence of established clinical competence/entrustment - Clinical workplace experiences: unpredictable environment; teaching non-standardized and “opportunistic” - Evidence of gaps in the competence of new medical graduates and associated patient safety concerns |
| 2. Targeted needs assessment  a. Targeted learners  b. Targeted learning environment | - Deficits in the clinical experiences (CoVID19 pandemic, structural deficits) - Workplace-based assessment data: students need focused high-yield skills training prior to graduation |
| 3. Goals and objectives  a. Broad goals  b. Specific measurable objectives | - Addressing all 13 EPAs as part of an intensive training program during TTR. - Addressing cognitive, psychomotor, and emotional skills for both individuals and teams. - Sessions LOs: granular, aligned with the EPAs and PLOs and the assessment tools. |
| 4. Educational strategies  a. Content  b. Methods  c. Faculty development | *Preparation*   - Framework: Kolb’s Experiential Learning, starting at the active experimentation phase, and Deliberate Practice - Simple skills (e.g., IM injection) and more complex skills (e.g., airway ventilation) are broken down into concrete steps for learners to follow - Criteria for competency: entrustment based on learners functioning fairly independently with minimal intervention by the experts (MOCAS) - Incorporating teaching on common errors related to various skills, and to prevent/rectify them (e.g., premature closure in clinical reasoning) - Building evidence-based content (e.g., updated IPSGs) - Involving SME in content development (e.g., anesthesia consultant determined the need to add US-guided central line insertion)   *Educational methods*   - Flipped classroom: pre-session reading + online exercises using LMS - Simulation-based education as the method to execute the LOs (e.g., escape room to teach on medical error detection and reporting) - Simulation Modalities (simple vs complex task trainers): multimodal, aligned with task complexity and fidelity/realism requirements; virtual simulation using Medscape Patient Case Simulations^48^ for NoC - Review of performance to competence benchmarks: by demonstrating 1^st^ to the students, then observing them practice under supervision   *Faculty development*   - Train the faculty on the use of assessment tools and LMS |
| 5. Individual assessment and feedback | *Development*   - Pre- and post-course self-confidence assessment in all relevant competencies - Direct observation of performance assessment - Assessment tools: included skill-specific tools such as the (POCUS)-specific global rating scale, and more generic skills rating scale but that also includes a form of reflection; e.g. DOPS + the Ottawa scale for assessment of entrustability completed by both the facilitators and the learners (self-assessment of entrustability - Documentation of other forms of validity evidence: currently following up with graduates on the extent to which they successfully transferred these skills into their residency training   *Use*   - Formative assessment with non-compensatory pass/fail judgment |
| 6. Program evaluation  a. Aggregated learner assessments  b. Assessment of curricular components, strengths and  areas for improvement  c. Assessment of the practical value of the course (does it accomplish its intended purpose?) | - All assessment data, including learner assessment data and sessions’ evaluations are collected and stored in LS - Plan to conduct long-term outcomes analysis of learner performance - Team reflects on evaluation results yearly - Evaluation results feed into the QI of the program |
| 7. Implementation  a. Political and administrative support  b. Resources  c. Administration of curriculum  d. Identification and address of barriers  e. Introduction of curriculum (piloting or phasing in) | - University support secured to launch the course in 2023 - Resources: both human and technological resources available at KU - Administration of the curriculum: overseen by a team of highly skilled simulation experts and with the support of technical experts in running the LMS - Piloting the curriculum: in 2023 - Barriers: identified and included in the post-course reflections/debriefing session, and alternative approaches adopted. For example, the workload was not properly estimated in the pilot phase, and thus a revised workload plan was applied afterwards. |

**Table 2. Blueprint of SLOs, PLOs, EPAs-competencies matrix, assessment tools, and the pre-post self-confidence assessment**

| Session | SLOs | EPA |  | Competency domains | PLOs |  | Assessment Tools | Pre-post test self-confidence Qs *“I feel confident in …”* on a 4-point Likert scale |
| --- | --- | --- | --- | --- | --- | --- | --- | --- |
| Session 1: Patient Safety | 1. Discuss the concept of patient safety and safety culture 2. Discuss the healthcare system and become the concept of human factors/ergonomics using clinical case scenarios 3. Define the steps that need to be taken when harm or near miss is identified 4. Define the roles of resident trainees in supporting and championing patient safety 5. Identify breaches in safety practices in a simulated environment 6. Define root cause analysis (RCA) and the most common tools to conduct a RCA 7. Using the RCA, report safety events using the appropriate channels and reporting pathways and develop an action plan to improve safety | EPA 13 |  | **KP1 ICS2 P4 PPD5**  **PBLI4 PBLI10** | 14, 15, 20, 22, 24 |  | Direct observation rating checklist of RCA | - Recognizing safety events of different types - Reporting safety events using the correct procedures and terminology - Participating in an RCA |
| Session 2: Intern and Resident Workday | 1. Recognize and discuss expectations of resident life, including routine activities and tasks (calling a consultant, admissions and discharges etc.) 2. Identify and locate useful resources for learning and balancing learning and work 3. Discuss the importance of recognizing one’s own limits and knowing when to call for help to maintain patient safety 4. Discuss tips and tricks to optimize functionality and wellbeing, with special focus on balancing family and parenting responsibilities with residency workload (address questions related to motherhood) | EPA 7      EPA 9      EPA 13 |  | **KP3 PBLI6 PBLI1 PBLI3**  **PBLI6 PBLI7**  **IPC2 SBP2 ICS3**  **ICS2/IPC3 IPC1 ICS7 P1**  **P4 SBP5** | 5, 14, 18, 22, 24 |  | None | - Identifying useful resources for learning and maintaining a healthy balance between life and work responsibilities |
| Session 3: Medication Safety Session | 1. Discuss the best practices for safe medication prescription, including inpatient medication order entry into electronic medical records (EMR). 2. Enter a correct medication order on LearningSpace based on 2 given clinical vignettes. | EPA 4        EPA 13 |  | **PC6 PBLI1**  **PC5 PC2**  **PBLI7**    **SBP4** | 3, 11, 15,17,20, 23, 24,25 |  | Peer assessment using rating checklist (MedEdPortal) | - Identifying the major/common medication safety errors - Entering a correct medication order |
| Session 4: BLS | 1. Provide high-quality CPR using mannequins (adult, children, and infant) 2. Explain the AHA Chain of Survival, specifically the BLS components 3. Recognize the important early use of an AED 4. Perform effective ventilations using a barrier device 5. Discuss the importance of teams in multirescuer resuscitation and performance as an effective team member during multirescuer CPR 6. Perform relief of foreign-body airway obstruction (choking) on mannequins (adult and infant) | EPAs 9  EPA 10              EPA 12 |  | **IPC2 SBP2 ICS3**  **PC2 PC4 PC5**  **PC4 PC3 PC2 PC5 PC6**  **PPD1**  **PC1 PPD1 SBP2 IPC4**    **PC1** | 1, 3, 5, 10, 22 |  | BLS rating rubric | - Performing CPR, including effective chest compressions and ventilation - Recognizing when to use AED - Removing a foreign body causing airway obstruction |
| Session 5: Procedural Skills Laboratory I:  IV & ABG collection;  IO & IM | 1. Demonstrate independently the proper preparation and steps to effectively perform the procedural skill, including tools, setting up and maintaining a sterile field, and safe disposal of contaminated tools and sharps. 2. Describe the indications, consent requirements, preparation and steps to accomplish these procedural skills. 3. Identify the different types of suturing 4. Perform skills successfully | EPA 12        EPA 13    EPA 14 |  | **PC1**  **PC7 ICS6 P6**  **PPD7 PPD1**    **SBP4**    **PBLI1 PBLI2** | 1, 3, 5, 6, 10, 12,21 |  | DOPS  Ottawa Scale          Modified Ottawa Scale (learner version) | - Inserting a peripheral IV line - Inserting a urinary catheter on adult female/male - Identifying when and how to insert IO in adults and children - Identifying when and how to do IM injection in adults and children - Perform various surgical suture materials and techniques |
| Session 6: Procedural Skills Laboratory II: LP, Informed Consent, POCUS | 1. Describe the required elements for informed consent, 2. Successfully obtain informed consent from a SP 3. Describe the indications, consent requirements, preparation and steps to perform a successful LP, including tools, patient positioning, setting up and maintaining a sterile field, opening pressure measurement, sample collection, and safe disposal of contaminated tools and sharps. 4. Perform point-of-care ultrasound under direct guidance of SME then independently. | EPA 12        EPA 13    EPA 14 |  | **PC1**  **PC7 ICS6 P6**  **PPD7 PPD1**    **SBP4**    **PBLI1 PBLI2** | 1, 3, 5, 6, 10, 12,21 |  | DOPS  Modified Ottawa Scale          Modified Ottawa Scale (learner version) | - Explaining and obtaining informed consent - Performing LP - Performing POCUS and identifying major structures in neck, joints, abdomen |
| Session 7: Basic & advanced airway management  + Mechanical ventilation | 1. Demonstrate how to effectively accomplish ‘bag and mask’ ventilation 2. Describe the indications and preparation for endotracheal intubation 3. Perform ET intubation on a PTT 4. Recognize a failed ETT 5. Describe the procedure of cricothyroidotomy 6. Review and report on the condition of a patient who is receiving mechanical ventilation | EPA 12  EPA 10 |  | **PC1**  **PC2 PC3 PC4 PC5 PC6**  **PPD1** | 3, 5, 7, 9 |  | In-house direct observation rating checklists | - Performing bag-n-mask ventilation - Describing the tools for and performing ET intubation |
| Session 8: ACLS (over 2 days) | 1. Identify key science that drives increased patient survival. 2. Apply the BLS, Primary, and Secondary Assessment sequence for a systematic evaluation of adult patients. 3. Perform prompt, high-quality BLS, including prioritizing early chest compressions and integrating early AED use. 4. Recognize and perform early management of respiratory arrest. 5. Demonstrate effective and safe use of manual defibrillator. 6. Discuss early recognition and management of acute coronary syndrome, including appropriate disposition. 7. Identify early recognition and management of stroke, including appropriate disposition. 8. Model effective communication as a member of a high-performance team. 9. Recognize the impact of team dynamic on overall team performance. 10. Recognize cardiac arrest. 11. Perform early management of cardiac arrest until termination of resuscitation or transfer of care. 12. Recognize bradyarrhythmias that may result in a cardiac arrest or complicated resuscitative outcome. 13. Perform early management of bradyarrhythmias that may result in cardiac arrest or complicate resuscitation. 14. Recognize tachyarrhythmias that may result in a cardiac arrest or complicated resuscitative outcome. 15. Perform early management of tachyarrhythmias that may result in cardiac arrest or complicate resuscitation. 16. Perform early management of cardiac arrest until termination of resuscitation or transfer of care, including immediate post-cardiac arrest care. 17. Demonstrate team member behaviors according to roles during megacode cases. | EPA 9            EPA 10 |  | **IPC2 SBP2 ICS3**  **ICS2/IPC3 IPC1 ICS7 P1**  **P1 ICS7 IPC1 SBP2**    **PC2 PC4 PC5**  **PC4 PC3 PC2 PC5 PC6**  **PPD1**  **PC1 PPD1 SBP2 IPC4** | 1, 3, 5, 6, 7, 10, 11,22 |  | ACLS Rating rubrics | - Distinguishing between the indications for AED vs defibrillator - Identifying early signs of ACS and stroke and initiating correct management - Differentiating between the different types of arrhythmias and initiating the correct management - Functioning as a megacode team member |
| Session 9: Procedural Skills III: Suturing;  Foley catheter insertion | 1. Demonstrate independently the proper preparation and steps to effectively perform the procedural skill, including tools, setting up and maintaining a sterile field (Foley), and safe disposal of contaminated tools and sharps. 2. Describe the indications, consent requirements, preparation and steps to accomplish these procedural skills. 3. Identify the different types of suturing   Perform skills successfully | EPA 12        EPA 13    EPA 14 |  | **PC1**  **PC7 ICS6 P6**  **PPD7 PPD1**    **SBP4**    **PBLI1 PBLI2** | 1, 3, 5, 6, 10, 12,21 |  | DOPS  Modified Ottawa Scale          Modified Ottawa Scale (Learner version) |  |
| Session 9: NOC | 1. Answer a pager call from a nurse as part of a night on call duties 2. Obtain interval history from nurse, patient, and EMR 3. Formulate a DDx that explains the patient’s symptoms 4. Derive evidence-based practice to address the working Dx while accounting for the patient’s past hx 5. Perform an oral presentation of the case to the on-call resident/consultant 6. Document an on-call-note in the EMR 7. Perform a handover process to your colleague | EPA 1    EPA 2            EPA 3              EPA 4        EPA 6        EPA 7              EPA 8          EPA 9 |  | PC2, KP1    **PC2 KP3 KP4 KP2**  **PC4 KP3 KP4 PPD8 PBL1**  **P3 KP4 ICS2**    **PC5 PC9 SBP3 PBLI9 KP1 KP4**  **PC5 PC7 KP1 KP4 SBP3 PBLI9**  **PC4 PC5 KP1**    **PC6 PBLI1**  **PC5 PC2**  **PBLI7 SBP3**    **PC2 PBL1 PPD4 P1**  **ICS2 PC6**    **KP3 PBLI6 PBLI1 PBLI3 PBLI6 PBLI7 PBLI1 PBLI8 PBLI9 PC7 PBLI1 PBLI8**  **PBLI9 PC7**    **PBLI7 ICS2 ICS3 P3 ICS2 ICS3 ICS2 PC8 PBLI5 ICS2 ICS3**    **IPC2 SBP2 ICS3** | 1, 3, 5, 6, 7, 8, 9, 10, 11, 12, 14,22 |  | Oral presentation rubric    IPASS | - Communicating with the nurse as an on-call PGY1 - Attending to acute changes in patient status as an on-call PGY1 - Performing OP to senior resident/faculty - Performing effective handover to peer |
| Session 11: Acute Case Management | 1. Evaluate Patients with Acute Changes in Condition in Respective Specialty (Surgery, Medicine, Pediatrics) 2. Determine the cause of the acute deterioration and initiate emergency management 3. Discuss the diagnosis and management, including fluid resuscitation, of patients with acute changes in condition. | EPA 2          EPA 3            EPA 10 |  | **PC2 KP3 KP4 KP2 PC4 KP3 KP4 PPD8 PBL1 KP3 KP4 ICS2**    **PC5 PC9 SBP3 PBLI9 KP1 KP4 PC5 PC7 KP1 KP4 SBP3 PBLI9 KP1**    **PC2 PC4 PC5 PC4 PC3 PC2 PC5 PC6 PPD1** | 1, 3, 5, 6, 7, 9, 10, 12, 22 |  | In-house Direct Observation Rating Checklist | - Identifying, evaluating and managing a patient with acute changes |

**Table 3.** Capstone course curricular reforms from 2023 to 2025

|  | Capstone 2023 *Pilot Phase* | Capstone 2025 *The Reenvisioned Curriculum* |
| --- | --- | --- |
| Student volume | 23 | 31 |
| Content Development | - Core EPAs 2, 3, 7, 8, 10-13 - Medication safety: CBL on the different stages of medication management and use, and the potential errors that can happen in each stage - Patient safety: Identify safety breaches + error reporting (RCA) - Handover and effective communication: standalone session - Basic airway management | - Core EPAs 1 through 13 - Medication safety: prescription and EHR order entry (CBL) - Patient safety: simulation-based activity “Escape Room” to apply knowledge of safety breaches + error reporting (RCA) - BLS (introduced in 2024) - ACLS (introduced in 2024) - NoC (introduced in 2024) - Handover removed as a standalone session and kept as direct application in NoC - Basic + Advanced airway management |
| Facilitators | - Mainly internal KUCMHS, with 15 externals | - Internal + 25 externals, including residents from affiliate hospitals (higher facilitator:learner ratio) |
| Assessment tools | - Proficient/ Not Proficient Checklist - Pre- and Post- Course Self-Confidence Survey by skills and competency domains | - DOPS + Modified Ottawa Co-Activity Scale (for both facilitator and student) - POCUS Assessment Tool - Weekly post-session evaluation - Pre- and Post- Course Self-Confidence Survey based on high-yield skills mapped to EPAs |
| Assessors | - Facilitators | - Assigned stand-alone assessors |
| Session Scheduling | - 11 sessions | - 13 sessions |
| Role assignment | - One faculty lead and one staff lead | - One faculty lead, two faculty co-leads, 2 staff leads, assigned roles and tasks clearly discernable |
| e-learning | - Limited to pre-session learning materials and short quizzes | - Used virtual simulation (Medscape Sim) for NoC |
| Digitization | - Limited to pre-session learning materials and short quizzes (Blackboard LMS) | - Fully digitized (paperless) learning (Blackboard LMS), in-class CBL, and assessment (LearningSpace LMS) |

**Table 4.** Typical capstone roster

| Capstone – 1^st^ Circuit – Advanced Procedural Skills II – April 14, 2025  10:00 AM – 01:00 PM | | | |
| --- | --- | --- | --- |
| Start Time | Station #1 (Body Interact)  POCUS – Abdominal &Cardiothoracic  Facilitators: Dr. SA, Dr. NA, Dr. YA, Dr. SA, Dr. AR  Assessors: Dr. SA, DL | Station #2 (Task-training Room 1)  POCUS – Central line insertion  Facilitators: Dr. PL  Assessors: AS | Station #3 (Task-training Room 2)  POCUS – Peripheral line insertion (arterial &venous)  Facilitators: Dr. OF  Assessors: IT |
| 10:00 AM | Group 1 | Group 3 | Group 2 |
| 10:30 AM | Group 2 | Group 2 | Group 3 |
| 11:00 AM | Group 3 | Group 1 | Group 1 |
| 11:30 AM | Group 4 | Group 6 | Group 5 |
| 12:00 PM | Group 5 | Group 4 | Group 6 |
| 12:30 PM | Group 6 | Group 5 | Group 4 |

**Table 5.** Excerpts from capstone course of academic year 2025 weekly sessions’ self-confidence survey results

| **main / Topic** | **Full Description** | **Competency Category** | **Strongly Agree** | | **Agree** | **Disagree** | **Strongly Disagree** | **No Response** | **Percentage positive** | **Percentage negative** |
| --- | --- | --- | --- | --- | --- | --- | --- | --- | --- | --- |
| **Patient Safety** | Training in safe clinical practice, error prevention, and minimizing patient risks. | Non-Technical Skill; Patient Safety/Quality | 24.19% | | 53.23% | 0.00% | 3.23% | 19.35% | 77.42% | 22.58% |
| **Resident Workday** | Exposure to the workflow, structure, and challenges of a resident’s clinical day. | Non-Technical Skill; Professionalism/Systems | 37.10% | | 40.32% | 0.00% | 3.23% | 19.35% | 77.42% | 22.58% |
| **Medication Safety Session** | Training in correct medication order entry into medical record | Non-Technical Skill; Patient Safety/Quality | 19.35% | | 54.84% | 3.23% | 3.23% | 19.35% | 74.19% | 25.81% |
| **Basic Airway Management** | Practical training in basic airway techniques (bag-mask, adjuncts) | Technical Skill; Acute Care Management | 37.10% | | 46.77% | 0.00% | 0.00% | 16.13% | 83.87% | 16.13% |
| **Advanced Airway Management** | Advanced techniques (intubation, supraglottic devices). | Technical Skill; Acute Care Management | 37.10% | | 46.77% | 0.00% | 0.00% | 16.13% | 83.87% | 16.13% |
| **Procedural Skills (IV, IM, IO, ABG)** | Hands-on training in IV line placement, intramuscular injection, intraosseous access, and arterial blood gas sampling. | Technical Skill; Procedural Competency | IV | 50.00% | 33.87% | 0.00% | 0.00% | 83.87% | 16.13% |  |
|  |  |  | IM | 53.23% | 30.65% | 0.00% | 0.00% | 83.87% | 16.13% |  |
|  |  |  | IO | 54.84% | 29.03% | 0.00% | 0.00% | 83.87% | 16.13% |  |
|  |  |  | ABG | 53.23% | 30.65% | 0.00% | 0.00% | 83.87% | 16.13% |  |
| **POCUS** | Training in point-of-care ultrasound for bedside diagnostics and procedural guidance. | Technical Skill; Acute Care/Diagnostics | 32.26% | | 54.84% | 0.00% | 0.00% | 12.90% | 54.84% | 12.90% |
| **Procedural Skills (Informed Consent, LP, Foley Catheter)** | Training in obtaining consent, lumbar puncture, and Foley insertion. | Mixed: Technical + Non-Technical (Procedural + Communication) | IC | 38.71% | 48.39% | 0.00% | 0.00% | 87.10% | 12.90% |  |
|  |  |  | LP | 35.48% | 51.61% | 0.00% | 0.00% | 87.10% | 12.90% |  |
|  |  |  | FC | 38.71% | 48.39% | 0.00% | 0.00% | 87.10% | 12.90% |  |
| **Night On Call** | Simulation of on-call duties including prioritization, triage, and acute problem solving. | Non-Technical Skill; Acute Care Management | 33.06% | | 52.42% | 1.61% | 0.00% | 12.90% | 52.42% | 14.52% |
| **Acute Case Management** | Managing acute presentations using reasoning, teamwork, and timely interventions. | Mixed: Technical + Non-Technical; Acute Care/Teamwork | 32.26% | | 54.84% | 0.00% | 0.00% | 12.90% | 54.84% | 12.90% |

**Table 6.** Workload distribution

|  |  |  | Number or personnel across the years | | |
| --- | --- | --- | --- | --- | --- |
| **Description** |  | **Capstone personnel** | 2023 | 2024 | 2025 |
| Designs, approves, and oversees all capstone operations including ensuring overall coherence as well alignment with EPAs (Dr. DM in 2023, Dr. ZD 2024 onwards) | **CD** | Course Director | 1 | 1 | 1 |
| Supports Course Director in design of program, including suggesting External SMEs | **ISME** | Internal SMEs | 1 | 2 | 2 |
| SMEs in the field that delivered the session but were not involved in design | **ESME** | External SMEs | 15 | 20 | 25 |
| Goes over all logistical requirements to implement sessions and communicates to all involved | **CC** | Course Coordinators | 1 | 1 | 2 |
| Set-up of rooms, simulators, equipment, etc. | **ST** | Simulation Team | 2-3 | 3-4 | 4 |
| Booking and training of SPs | **SPCT** | SP Coordinator & Trainer | 1 | 1 | 1-2 |
| Scheduling and recording of activities on LS | **LSC** | Learning Space Coordinator | 1 | 1 | 1 |

| **Phase** & General Activity | **Approximate time spent (mins)** | **Frequency (estimation)** |
| --- | --- | --- |
| **Planning Activities** | | |
| Weekly meetings (from Fall semester until completing the capstone course) | 30-60 | 20 |
| **Preparation Activities** | | |
| Initiating contact with all ESMEs | 30 | Equal to number of ESMEs |
| Processing security clearances | 30 | Equal to number of ESMEs |
| Initiating contact with students (including needs assessment survey) + follow ups | 240 | 1 |
| Preparing modules for students (on BB) | 60 | number of sessions |
| Developing schedules/ sequence of groups | 60 | number of sessions |
| **Implementation Activities** | | |
| Set-up of session by ST | 60 | number of sessions |
| Communication of session information to all students and instructors by CC | 30 | number of sessions |
| Running each session | 180 (minimum) | number of sessions |
| Evaluation surveys and extraction of results | 40 | number of sessions |

**Table 7.** A comparison of percentages of students’ satisfaction within capstone course sessions (class of 2023 vs Class of 2025).

| 2023 Capstone course evaluation  (n=23 students) | | Strongly agree | | | Agree | | Disagree | Strongly disagree | |
| --- | --- | --- | --- | --- | --- | --- | --- | --- | --- |
| Course's contribution to students' skills, knowledge and communication | | 58.33% | | | 39.81% | | 1.85% | 0.00% | |
| Preparedness and quality of delivery | | 58.52% | | | 40.00% | | 1.48% | 0.00% | |
| Satisfaction with course | | 44.44% | | | 51.85% | | 3.70% | 0.00% | |
| 2025 Capstone course evaluation  (n=31 students) | | | | | | | | | |
| Week | **Session** | | **Strongly agree** | **Agree** | | **Disagree** | | | **Strongly disagree** |
| Week 1 | Patient Safety Session | | 32.69% | 63.46% | | 0.00% | | | 3.85% |
|  | Resident and Intern Workday | | 48.08% | 48.08% | | 0.00% | | | 3.85% |
|  | Medication Safety Session | | 26.92% | 65.38% | | 3.85% | | | 3.85% |
| Week 2 | Basic and Advanced Airway Management | | 46.30% | 53.70% | | 0.00% | | | 0.00% |
|  | Procedural Skills (IV, IM, IO & ABG withdrawal) | | 64.35% | 35.65% | | 0.00% | | | 0.00% |
| Week 3 | POCUS | | 37.04% | 62.96% | | 0.00% | | | 0.00% |
|  | Procedural Skills (Informed Consent; Lumbar Puncture; Foley Catheter Insertions) | | 43.21% | 56.79% | | 0.00% | | | 0.00% |
|  | Night On-Call | | 37.96% | 60.19% | | 1.85% | | | 0.00% |
|  | Acute Case Management | | 37.04% | 62.96% | | 0.00% | | | 0.00% |

*Appendix 1. KUCMHS IRB Approval*

**

*Appendix 2. KUCMHS MD Program Learning Objectives*


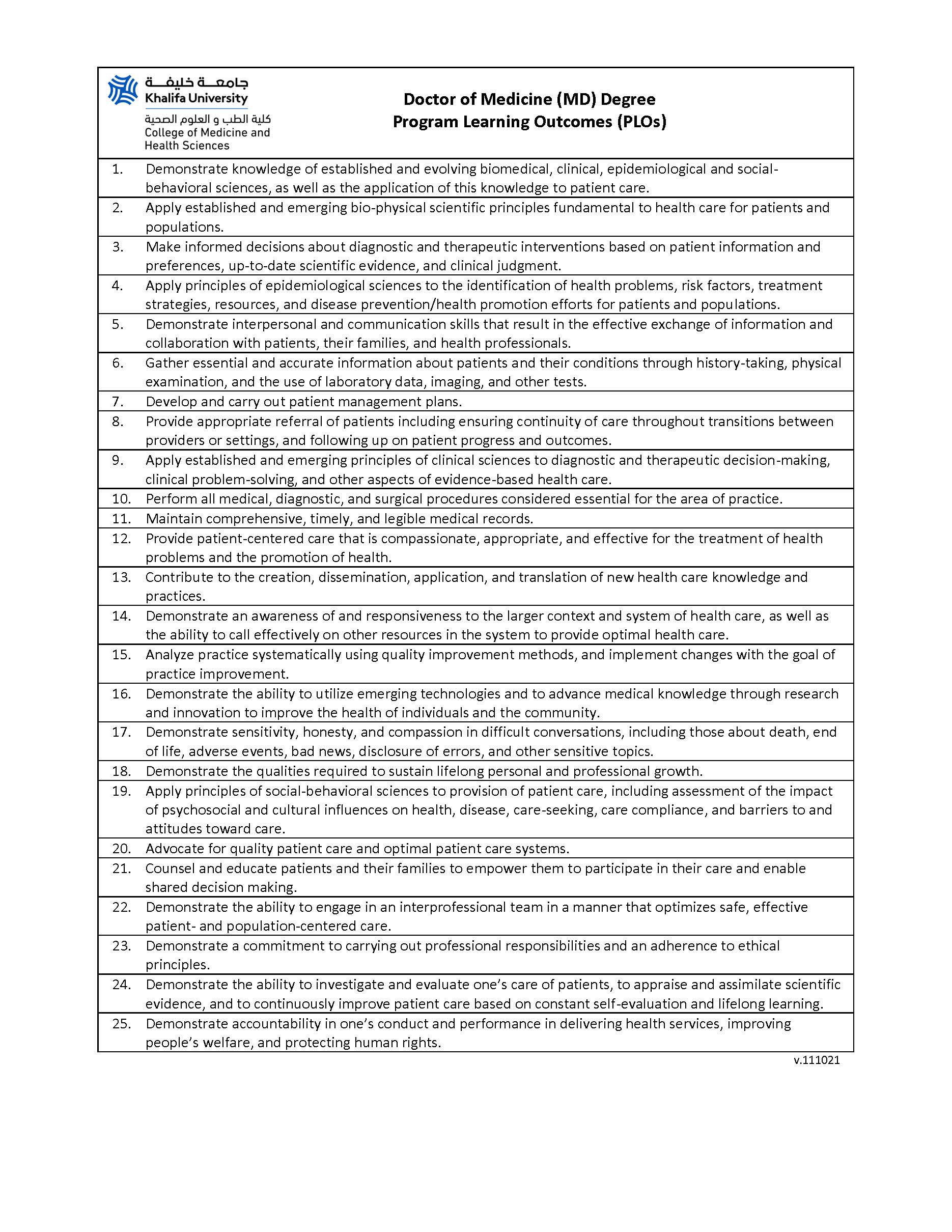


*Appendix 3. Sample of DOPS with MOCAS from the Foley catheter station extracted from LearningSpace™. (a) Trainees post-station self-assessment of entrustment (average for all students shown) and (b) Facilitator post-station assessment of entrustment (average for all students shown)*

a

b
